# Th2 Biased Immunity With Altered B Cell Profiles in Circulation of Patients With Sporotrichosis Caused by *Sporothrix globosa*

**DOI:** 10.3389/fimmu.2020.570888

**Published:** 2020-11-13

**Authors:** Jianjiao Zu, Lei Yao, Yang Song, Yan Cui, Mengqi Guan, Ruili Chen, Yu Zhen, Shanshan Li

**Affiliations:** Department of Dermatology and Venerology, The First Hospital of Jilin University, Changchun, China

**Keywords:** sporotrichosis, *Sporothrix globosa*, immunity, T cells, B cells

## Abstract

Sporotrichosis is a subcutaneous mycotic infection, and *Sporothrix* *globosa* is one of the causative agents with a worldwide distribution, notably in Asia. However, the immune profile in human sporotrichosis caused by *S. globosa* still remains obscure. Here, we demonstrated enhanced Th2 response in circulation with significant increases in Th2 frequency, Th2/Tregs as well as IL-4 seretion in patients. Elevated IL-17A^+^Th17 percentage was accompanied with reduced IL-17A level in serum, which may imply a dysfunction of this CD4^+^T subset in *S. globosa* infection. In addition, Th2 percentage, the ratios of Th2/Tregs and Th17/Tregs were all raised in patients with fixed cutaneous form, while only Th2/Tregs displayed increment in lymphocutaneous form. Meanwhile, the percentage of double negative B cells was significantly increased and positively correlated with Th2 and Tregs in whole patients. Except naïve B cells, all memory B cells together with Th2 cells increased in patients with short duration (less than 6 months), which may suggest a collaboration of T cells with altered B cell profile in human sporotrichosis caused by *S. globosa*. In consistent with the changes of IFN-γ^+^Th1, IL-4^+^Th2 and IL-17A^+^Th17 in patients with short duration, the percentages of these effector T cells all expanded when cocultured with *S. globosa* yeast cells in vitro. These data shed light on the potential involvement of peripheral T and B cell immunity against this mycotic infection and indicated that different immune responses existed in different stages of sporotrichosis; meanwhile different immune profile may contribute to different clinical manifestations of this disease.

## Introduction

Sporotrichosis is a subcutaneous mycotic infection caused by *Sporothrix* complex, among which *Sporothrix schenckii* sensu stricto (*S. schenckii*), *Sporothrix brasiliensis* (*S. brasiliensis*), and *Sporothrix globosa* (*S.* *globosa*) are most clinically relevant. *S. globosa* has a global distribution evidenced by isolates from Europe (the United Kingdom, Spain, and Italy), the United States, South America (Mexico, Guatemala, and Columbia), and Asia (India, China, and Japan) (1–6). Compared with other species of *Sporothrix* complex, the clinical manifestations caused by *S.* *globosa* are relatively mild owing to its low to moderate virulence (1, 7–9), which indicates it may trigger different immune responses in the host.

Up to now, the defense mechanisms against *Sporothrix* complex in human are very limited. In mice, previous studies have acquired a consensus that T cell-mediated immunity particularly Th1 response is considered critical to host defense against *S. schenckii* infection (10, 11) which participates in the granuloma formation in skin lesions (12) and enhances phagocytosis of the pathogen by macrophages (13, 14). Besides Th1, evidence suggested that control of *S. schenckii* is Th17 dependent (12, 15). Th17 response is found crucial for optimal *S. schenckii* clearance (16) and the host tends to use a polarized Th17 response when phagocytosis capacity is impaired in vivo (17). So far, only one published literature mentioned that great reduction in Th1 and Th17 was found in patients with sporotrichosis caused by *S. globosa* (18).

In addition to cellular immunity, humoral response in experimental sporotrichosis in mice has also been evaluated (19). IgG antibodies against *Sporothrix* antigens could be detected throughout the infection in mice (20). Anti-Gp70 is one of the antibodies that has been widely recognized as playing protective role against *S. schenckii* and *S. brasiliensis* infection (21, 22). Recently, anti-enolase antibodies were also characterized involved in antifungal responses in *S. schenckii* and *S. brasilensis* infected mice (23, 24). In human sporotrichosis, the IgG, IgM and IgA antibodies were detected in all of the clinical forms (25).

Despite the progress in the knowledge of humoral response in mice, little is known about the humoral immunity against clinically relevant *Sporothrix* species, especially *S. globosa* in human (1, 19). Follicular helper T cells (Tfh), a newly discovered CD4^+^ T cell subgroup, bridges the cellular and humoral immunity by assisting B cells’ differentiation and antibody production (26). However, in the host defense against *Sporothrix* complex, the change of B cells and Tfh cells and the mechanism driving the production of antibodies are still not clear.

To elucidate these questions, we investigated the immune profile in circulation by application of phenotyping circulating cells and cytokine detection in sera as well as cells-pathogen coculture in vitro. We showed a Th2 predominant response together with altered B cell profile in whole patients. Moreover, double negative B cells (IgD^-^CD27^-^ B cells) were positively correlated with Th2 and Tregs which indicated that B and T cells collaborated in the immunity against this pathogen. An elevated ratio of Th17/Tregs was noticed in patients with fixed form, not in patients with lymphocutaneous type. Except the decrease in IgG2, we did not find any change in the level of IgE, total IgG and IgM as well as the proportion of Tfh cells in patients, though CD27^+^ memory B subsets responsible for antibodies production were greatly changed.

## Materials and Methods

### Subjects

Sixty-eight patients with sporotrichosis (SP) and 45 age and sex matched healthy controls (HC) were recruited in this study. The definitive diagnosis of sporotrichosis was established based on clinical manifestation, skin pathology, and positive fungal culture. Isolates from patients were identified as *S. globosa* based on morphological characteristics and calmodulin-encoding gene (CAL) sequencing. All patients had not been treated with any drugs and had no history of any auto-immune or infectious diseases by asking questions about their symptoms and medical history. Patients enrolled were divided into subgroups according to their duration (till the moment of diagnosis) and clinical types respectively: shorter duration (SD) (< 6 months) and longer duration (LD) (> 6 months); fixed cutaneous form (FF) and lymphocutaneous form (LF). The detail information was listed in **Tables 1**, **2**, and **S1**. Written informed consents from all subjects were obtained. The study was approved by the First Hospital of Jilin University Research Ethics Committee.

**Table 1:** Demographic characteristics of sporotrichosis patients and healthy control.

	total patients (n = 50)	Disease duration		Presentation		HC (n = 25)
		duration <6 mon(n = 24; n_FF_ = 12, n_LF_ = 12)	Duration >6 mon(n = 26; n_FF_ = 21, n_LF_ = 5)	fixed form (n = 33; n_SD_ = 12, n_LD_ = 21)	lymphocutaneousform (n = 17; n_SD_ = 12, n_LD_ = 5)	
**Age** **(years, mean±SD)**	52 ± 13	48 ± 13	55 ± 12	51 ± 11	52 ± 15	53 ± 14
**Female/Male**	34/16	14/10	20/6	25/8	9/8	18/7
**Average duration (months, mean±SD)**	6.6 ± 5.2	2.6 ± 0.97	10.2 ± 4.8	7.6 ± 5.8	4.6 ± 3.2	–

The patients were No.1 to No.50 enrolled before 2020.07.10.

**Table 2:** Clinical characteristics of patients enrolled.

Patient no	Sex	Age	Clinical form	Disease duration(month)	Location	Previous history
1	F	48	Fixed	6	Face	
2	F	57	Fixed	3	Face	
3	M	61	Fixed	6	Upper limbs	
4	M	46	Fixed	2	Lower limbs	
5	F	50	Fixed	2	Upper limbs	
6	M	70	Fixed	1	Face	
7	F	48	Fixed	2	Upper limbs	
8	F	48	Fixed	12	Chest	
9	M	68	Lymphocutaneous	4	Face	
10	F	59	Lymphocutaneous	3	Upper limbs	
11	F	65	Fixed	8	Face	
12	F	51	Fixed	12	Face	hypertension
13	F	52	Fixed	6	Face	
14	F	42	Fixed	24	Upper limbs	
15	F	47	Lymphocutaneous	7	Face	
16	M	74	Lymphocutaneous	6	Upper limbs	hypertension
17	M	65	Lymphocutaneous	12	Face	hypertension
18	M	73	Lymphocutaneous	4	Upper limbs	
19	M	47	Lymphocutaneous	4	Face	
20	F	66	Fixed	10	Upper limbs	
21	F	81	Fixed	12	Face	
22	F	52	Lymphocutaneous	2	Neck	
23	F	75	Lymphocutaneous	12	Face	hypertension
24	M	48	Fixed	3	Face	
25	F	18	Fixed	2	Face	ointment
26	F	40	Fixed	3	Face	ointment
27	F	39	Fixed	7	Face	
28	F	63	Fixed	12	Upper limbs	
29	M	50	Fixed	7	Face	
30	F	30	Lymphocutaneous	2	Upper limbs	
31	F	52	Fixed	24	Face	
32	F	52	Lymphocutaneous	6	Upper limbs	ointment
33	F	46	Fixed	4	Face	
34	F	50	Fixed	9	Face	
35	F	54	Fixed	2	Face	
36	F	40	Lymphocutaneous	2	Upper limbs	
37	M	29	Lymphocutaneous	4	Face	
38	F	44	Fixed	12	Chest	
39	F	30	Lymphocutaneous	1	Face	
40	F	56	Fixed	6	Face	
41	M	48	Fixed	12	Face	
42	M	46	Lymphocutaneous	2	Face	
43	F	47	Fixed	12	Face	
44	F	37	Fixed	2	Face	
45	M	60	Fixed	1	Face	
46	M	53	Lymphocutaneous	3	Upper limbs	hypertension
47	F	53	Fixed	12	Back	
48	F	60	Fixed	7	Upper limbs	
49	M	50	Fixed	6	Upper limbs	
50	F	51	Lymphocutaneous	3	Upper limbs	
51	F	65	Lymphocutaneous	4	Face	
52	M	32	Fixed	10	Chest	
53	M	51	Fixed	6	Face	
54	M	77	Fixed	1	Face	
55	M	65	Fixed	6	Upper limbs	
56	F	65	Fixed	2	Upper limbs	Oral itraconazole
57	F	54	Fixed	6	Face	
58	M	45	Fixed	3	Upper limbs	
59	F	46	Fixed	12	Face	
60	M	52	Fixed	12	Lower limbs	
61	F	66	Lymphocutaneous	4	Upper limbs	
62	M	53	Fixed	6	Upper limbs	
63	F	73	Fixed	6	Upper limbs	
64	F	48	Lymphocutaneous	6	Face	
65	M	64	Fixed	4	Upper limbs	
66	F	79	Fixed	12	Upper limbs	
67	F	41	Fixed	6	Upper limbs	
68	F	36	Fixed	36	Neck	

No.51 to No.68 subjects were included in from 2020.07.10.

### Multiparametric Flow Cytometry Analysis

Peripheral blood mononuclear cells (PBMCs) were freshly isolated from heparinized venous blood by density gradient centrifugation on Ficoll-Lympholyte (Cedarlane Laboratories Limited, Ontario, Canada). Cells were stained with antibodies specific to surface markers at 4°C for 30 min. For intracellular staining, cells were further fixed, permeabilized and incubated with antibodies according to the manufacturer’s protocol. For intracellular detection of IFN-γ, IL-4, and IL-17A, freshly isolated PBMCs were first stimulated with Protein Transport Inhibitor (BD GolgiPlug containing Brefeldin A, BD Biosciences, USA) for 4 h in vitro. Unlabeled cells were used as autofluorescence control and IgG isotypes of corresponding antibodies were used as non-specific fluorescence control. FACS analysis was performed with a FACS Calibur flow cytometer (BD Biosciences, US). A total of 3×10^4^-5×10^4^ events for a selected gate (PBMCs gate) were collected for each sample and the data were analyzed by the FlowJo software (version 7.6). All antibodies used were listed in **Table 3**.

**Table 3:** Antibodies and fluorescent reagents used in flow cytometry.

	Antibody	Fluorochrome	Vendor	No./Clone
**Th cells**	Anti-human CD4	FITC	eBioscience	11-0049-42/RPA-T4
	Anti-human T-bet	PerCP-Cy5.5	eBioscience	45-5825-80/eBio4B10
	Isotype mIgG1	PerCP-Cy5.5	eBioscience	45-4714-82
	Anti-human GATA-3	APC	eBioscience	50-9966-41/TWAJ
	Isotype rIgG2b	APC	eBioscience	50-4031-82
	Anti-human ROR-ɣt	PE	eBioscience	12-6988-80/AFKJS-9
	Isotype rIgG2a	PE	eBioscience	12-4321-80
	Anti-human IFN-γ	PerCP-Cy5.5	eBioscience	45-7319-41/4S.B3
	Isotype rIgG2b	PerCP-Cy5.5	eBioscience	45-4031–80
	Anti-human IL-4	APC	eBioscience	17-7049-42/8D4-8
	Isotype mIgG1	APC	eBioscience	17-7049-42
	Anti-human IL-17	PE	eBioscience	12-7178-42/64CAP17
	Isotype mIgG1	PE	eBioscience	12-4714-82
**Tregs**	Anti-human CD4	FITC	eBioscience	11-0049-42/RPA-T4
	Anti-human CD25	PE	eBioscience	12-0259-41/BC96
	Anti-human CD127	APC	eBioscience	17-1278-41/eBioRDR5
**B cells**	Anti-human CD19	APC	eBioscience	17-0199-41/HIB19
	Anti-human IgD	FITC	eBioscience	11-9868-41/IA6-2
	Anti-human CD27	PE	eBioscience	12-0271-81/LG.7F9
**Tfh cells**	Anti-human CD4	PerCP-Cy5.5	eBioscience	35-0047-41/SK3
	Anti-human PD1	PE	eBioscience	12-2799-41/eBioJ105
	Anti-human CXCR5	FITC	eBioscience	11-9185-41/MUSUBEE

### ELISA

Serum levels of total IgG, IgG subclasses (IgG1, IgG2, IgG3 and IgG4), IgM, IgE, and T cell cytokines (IFN-γ, IL-4, IL-17A, and TGF-β1), respectively were determined using Invitrogen human uncoated ELISA Kit (Bender MedSystems GmbH, Campus Vienna BIocenter 2,1030 Vienna, Austria). For Ig measurement, we prediluted the samples in Assay Buffer according to the manufacturer’s instructions. The values of OD were detected by spectrophotometric instrument at a wave length of 450 nm.

### Strain and Co-Culture of PBMCs With *S. globosa*

The *S. globosa* strain was *Sporothrix globosa* ATCC4912, and yeast cells were grown in Brain Heart Infusion Agar (BD Bioxon) for 5–7 days at 37°C. After centrifugation at 3,000 r/min for 10 min, the yeasts were washed twice with phosphate-buffered saline (PBS), and then were resuspended in complete RPMI 1640 medium (supplemented with 10% fetal bovine serum and 1% penicillin/streptomycin). 2×10^5^ freshly isolated PBMCs were seeded in one well of 96-well plate and challenged with *S. globosa* yeast cells at different ratios (1:1; 1:2.5; 1:5; and 1:10). The coculture at each ratio had triplicates. 3 days later, PBMCs were restimulated with Protein Transport Inhibitor for additional 4 h. After restimulation, cells were harvested and stained with surface and intracellular mAbs as mentioned above.

### Statistical Analysis

All statistical analysis was performed using GraphPad Prism 5.01 (GraphPad software Inc, San Diego, CA, USA). The difference among three groups was analyzed with one-way ANOVA with Bonferroni correction. Unpaired t-test (Welch corrected) was utilized to analyze the data between two groups. The data was presented as mean±SD and *P* value <0.05 was considered significant.

## Results

### Th2 and Th17 Inclined Responses in Peripheral Blood of Sporotrichosis Patients Infected With *S. globosa*

**Figure 1:**
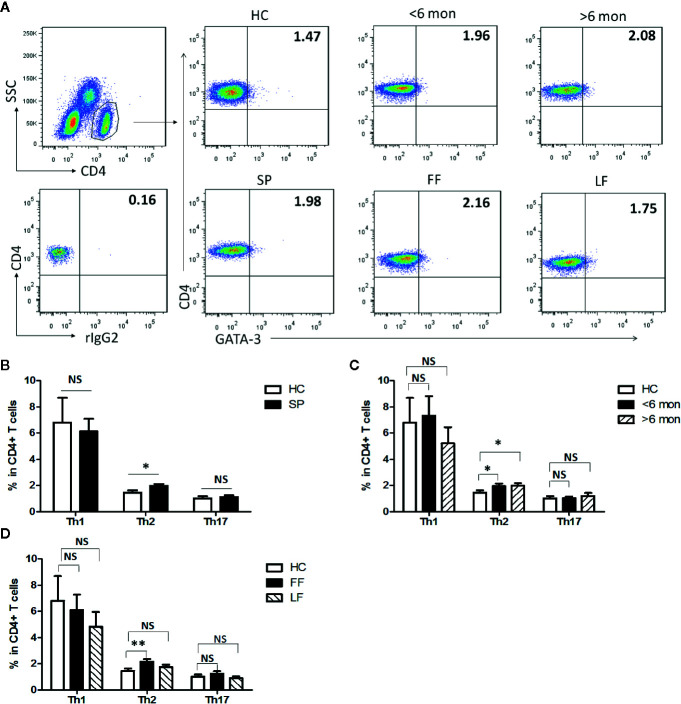
Altered distribution of Th1, Th2, and Th17 in PBMCs of patients. **(A)** PBMCs were stained intracellularly with T-bet, GATA-3, and ROR-γt mAbs after surface staining of CD4 mAb. According to CD4 staining and SSC, CD4^+^ T cells were gated. The parameters shown in quadrants of the representative graphs are mean frequency of GATA-3 for each group. **(B–D)** The average percentages of CD4^+^T-bet^+^ Th1, CD4^+^GATA-3^+^ Th2, CD4^+^ROR-γt^+^ Th17 are compared between HC (n = 24) and whole patients (n = 50) as well as subgroups of patients (SD, n = 24; LD, n = 26; FF, n = 33; LF, n = 17). Error bars represent mean±SD. **P < 0.01, *P < 0.05, and NS *P* ≥ 0.05.

Previous studies have suggested that T cell-mediated immunity plays an essential role in the immunity against infection with *S. schenckii* and *S. brasiliensis* (19). To make clear T cells’ profile in *S. globosa* infection in human, effector CD4^+^ T (Teff) cells were evaluated in SP (patients with sporotrichosis) and HC (**Figures 1**, **S1**). We analyzed the expressions of lineage specific transcription factors (T-bet, GATA-3, and ROR-γt) and cytokines (IFN-γ, IL-4, and IL-17A) in CD4^+^ T cells for the detection of Th1, Th2, and Th17 cells, respectively (**Figures 1A**, **S1A**). Interestingly, the percentage of CD4^+^GATA-3^+^ Th2 cells was significantly higher in whole patients than that of HC (*P* = 0.01, n = 49) (**Figure 1B**) which was further confirmed by the greatly increased frequency of CD4^+^IL-4^+^ Th2 cells (*P* < 0.0001, n = 18) (**Figure S1B**). Moreover, the proportion of Th2 cells remained at higher level throughout the course of disease as evidenced by its great increase both in the patients with short duration (SD) and long duration (LD) (*P* = 0.04, n = 24 and *P* = 0.02, n = 25 for GATA3^+^Th2; *P* < 0.0001, n = 7 and *P* = 0.0018, n = 11 for IL-4^+^Th2) (**Figures 1C**, **S1C**). In addition, similar increment was also observed in patients with fixed cutaneous form (FF) (*P* = 0.005, n = 16) rather than with lymphocutaneous form (LF) (**Figure 1D**).

It had been reported that Th1 response dominated in the immunity against infection with *S. schenckii* and *S. brasiliensis* (11). However, neither assessed by CD4^+^T-bet^+^ nor CD4^+^IFN-γ^+^ as definition of Th1 cells did we find statistical change in the proportion of this subset in whole patients compared with HC (**Figures 1B**, **S1B**). Meanwhile, we observed the dynamic change of Th1 cells in patients with different duration and found that only CD4^+^IFN-γ^+^ Th1 frequency had a significant elevation in SD (n = 7, *P* = 0.0006) while it dropped down in LD compared with HC (**Figures 1C**, **S1C**). No statistical difference was found among HC and patients with FF and LF (**Figure 1D**).

Besides, evidence suggested that control of *S. schenckii* is Th1/Th17 dependent (27) and optimal fungal clearance depends on an intact Th17 response during the *S. schenckii* systemic infection in mice (16). Similarly, in our study, we found that the frequency of CD4^+^IL-17A^+^ Th17 cells was greatly elevated in whole patients (*P* < 0.0001, n = 18) and remained high level in both patients with SD (*P* < 0.0001, n = 7) and LD (*P* < 0.0001, n = 11) (**Figures S1B, C**). However, CD4^+^RORγt^+^ Th17 cells had no change in whole patients and in patients with different duration or clinical types (**Figures 1B–D**).

### Imbalance Between Effector T Cells and Immunosuppressive T Cells in PBMCs of Sporotrichosis Patients

**Figure 2:**
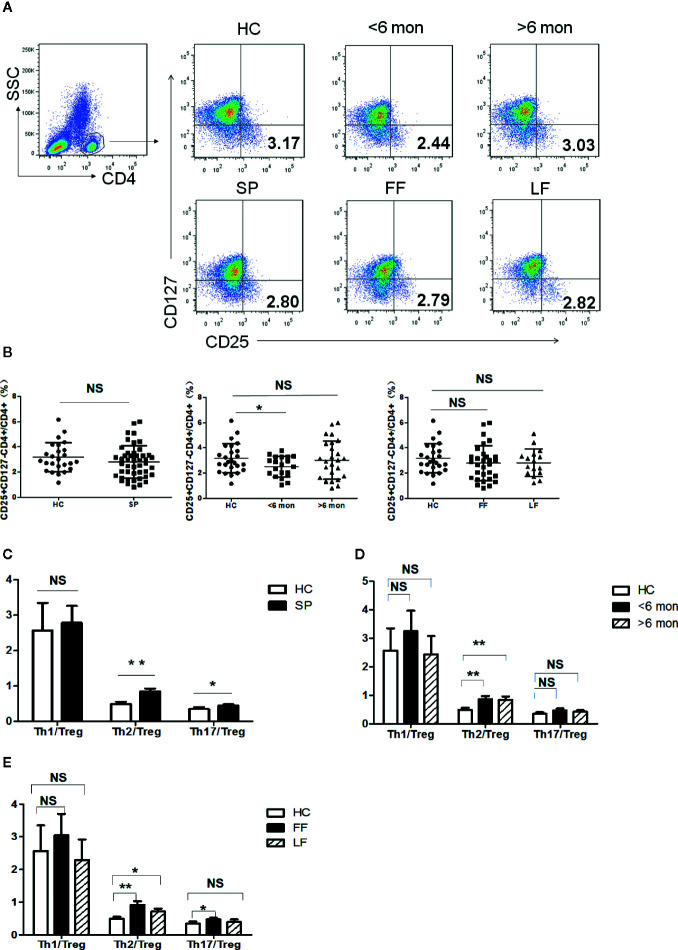
Tregs’ frequency and the ratio of effector T cells/Tregs in PBMCs of patients and HC. **(A)** PBMCs were stained with CD4, CD25 and CD127 mAbs. CD4^+^ T cells were gated according to CD4 staining and SSC and the proportion of CD4*^+^*CD25*^+^*CD127*^-^* Tregs in CD4*^+^* T cells were determined. The graphs are representative for patients, indicated subgroups and HC. Mean value of Tregs’ percentage is shown in each target quadrant. **(B–E)** Statistical graphs for comparing Tregs’ percentage and Th/Tregs ratios of the whole patients and indicated subgroups with HC. (SP, n = 46; SD, n = 20; LD, n = 26, FF, n = 30; LF, n = 16; HC, n = 25). Error bars represent mean±SD. **P < 0.01, *P < 0.05, and NS *P* ≥ 0.05.

Tregs are potent immunosuppressive CD4^+^ T cells which keep the balance of immunity. The ratios of Teff cells (Th1, Th2, Th17)/Tregs can better reflect the inclination of T cell-mediated immunity. We next analyzed CD4^+^CD25^+^CD127^-^ Tregs in the subjects’ peripheral blood (**Figure 2A**). As shown in **Figure 2B**, Tregs frequency showed no statistical change in whole patients and patients with different clinical types. However, a significantly decreased percentage of Tregs was found in SD group (*P* = 0.03, n = 20) compared with HC; while in LD, it showed a trend towards normalization.

Remarkably, Th2 inclined immune response was evidenced again by significant elevation in the ratio of Th2/Tregs not only in whole patients (*P* = 0.001, n = 46) but also in patients with different duration and clinical types (SD, *P* = 0.005, n = 20; LD, *P* = 0.006, n = 26; LF, *P* = 0.03, n = 16; and FF, *P* = 0.001, n = 30) (**Figures 2C–E**). In contrast to Th2 response, the ratio of Th1/Tregs kept statistically comparable to HC in patients regardless of the difference in duration and clinical types (**Figures 2C–E**). Besides, higher ratio of Th17/Tregs was found in whole patients (*P* = 0.04, n = 46) and in FF subgroup (*P* = 0.01, n = 30) (**Figures 2C, E**), which may suggest the participation of Th17 reaction in the formation of FF.

### Altered Distribution of CD19^+^ B Cell Subsets in Circulation of Sporotrichosis Patients

**Figure 3:**
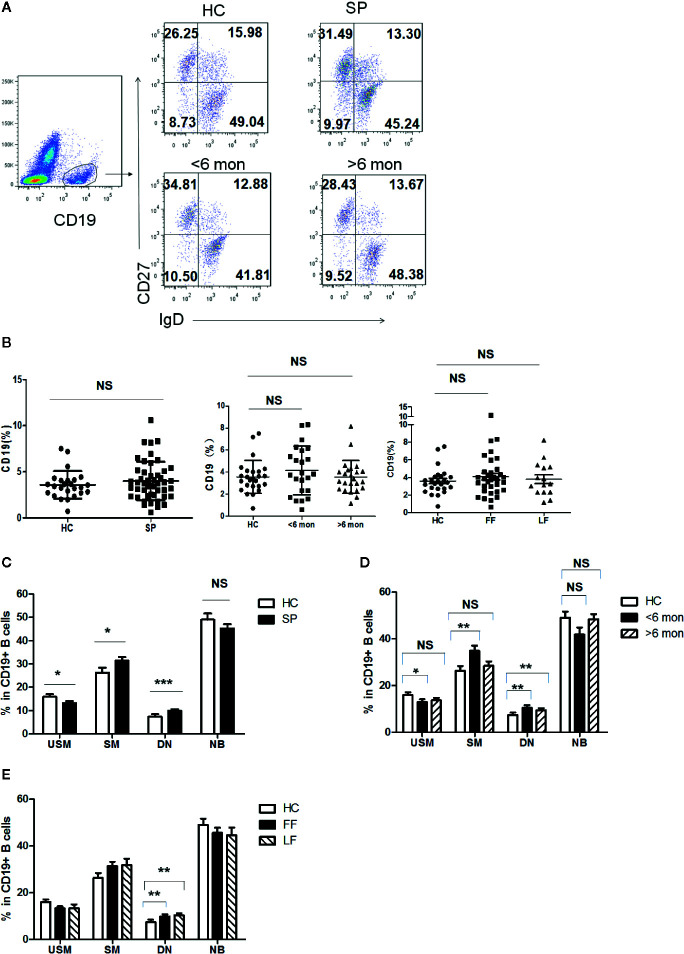
Phenotypic characterization of circulating B cell subsets in patients and HC. **(A)** Gated by CD19^+^ B cells, IgD^+^CD27^-^ NB, IgD^+^CD27^+^ USM B cells, IgD^-^CD27^+^ SM B cells, and IgD^-^CD27^-^ DN B cells were identified. The graphs are representative for HC and patients with different duration. Mean value of each B cell subset’s percentage is shown in the quadrants. **(B)** Statistical graphs for comparison of CD19^+^ B cells’ percentages between patient groups and HC. **(C–E)** Statistical graphs for distinct CD19^+^ B cells subsets between patients (SP, n = 48; SD, n = 23; LD, n = 25; FF, n = 33; LF, n = 15.) and HC (n = 25). Error bars represent mean±SD. *P < 0.05, **P < 0.01, ***P < 0.001, and NS *P* ≥ 0.05.

Previous reports indicated that humoral immunity also participate in the antifungal response in *S. schenckii* and *S. brasiliensis* infection (28). To figure out whether B cell populations are involved in the defense against *S. globosa* infection, we determined the profile of circulating CD19^+^ B cells in patients and HC. According to the expression of CD27 and IgD, B cells were divided into four subsets (**Figure 3A**): IgD^+^CD27^-^ naïve B cells (NB cells), IgD^+^CD27^+^ unswitched memory B cells (USM B cells), IgD^-^CD27^+^ switched memory B cells (SM B cells), and IgD^-^CD27^-^ double negative B cells (DN B cells). Although the frequency of total CD19+ B cells was unchanged in patients (**Figure 3B**), remarkably altered distribution of B cell subsets in whole patients (n = 48) was found (**Figure 3C**): a great reduction of USM B cells’ percentage (*P* = 0.03), significant increase of SM B cells (*P* = 0.03) and DN B cells (*P* < 0.001). Meanwhile, we noticed a similar variation in patients with SD (*P* = 0.03, *P* = 0.007, *P* = 0.008, respectively) (**Figure 3D**). However, in patients with LD, only DN B subset presented a significant elevation (*P* = 0.001). Patients with FF and LF both demonstrated enhancement in DN B cells (*P* = 0.007, *P* = 0.001, respectively) (**Figure 3E**). The frequency of naïve B cells remained comparable to that of HC in whole patients and patients with different duration and clinical types.

### Unchanged Tfh Frequency and Decreased IgG2 in Sporotrichosis Patients

**Figure 4:**
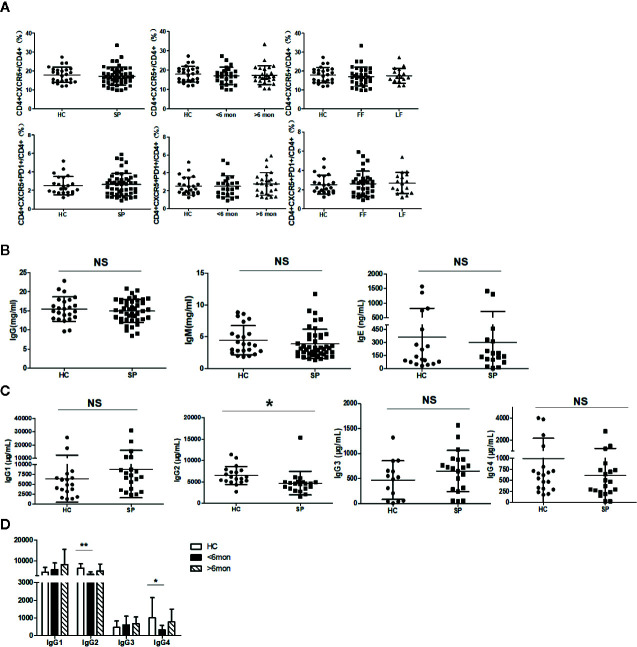
Unchanged cTfh cells and altered Ig profile in sporotrichosis patients. **(A)** Statistical graphs in the upper row were for CD4^+^CXCR5^+^Tfh while the lower row were for CD4^+^CXCR5^+^PD1^+^Tfh. The comparison of cTfh percentages were between HC (n = 24) and whole patients (n = 50), patients with different duration (< 6 mon, n = 24; > 6 mon, n = 26) and clinical types (FF, n = 33; LF, n = 17). **(B)** Comparison of serum levels of total IgG, IgM, and IgE between patients (n = 46) and HC (n = 24). **(C, D)** Distribution of IgG subtypes (IgG1, IgG2, and IgG3 and IgG4) in patients (in whole: n = 20; SD: n = 7; LD: n = 13) and HC (n = 19). Error bars represent mean±SD. *P < 0.05, **P < 0.01, and NS *P* ≥ 0.05.

It was reported that Tfh cells play a critical role in helping B cells differentiation and antibody production (29). To clarify whether the alteration of B cell subsets was associated with Tfh cells, we next investigated Tfh cells in circulation (cTfh). Compared with HC, the percentage of cTfh cells showed no statistical change in patients (**Figure 4A**).

Early studies have revealed that USM B and SM B cells are mainly responsible for IgM and IgG secretion respectively (30) and Th2 cells are widely recognized as IgE inducer. Considering significant changes in B cells and Th2 cells in our study, we next measured the concentration of IgG, IgM, and IgE in sera of the subjects. However, compared with HC, the levels of total IgG, IgM, and IgE had no significant changes in whole patients (**Figure 4B**) and patients with SD, LD, FF, and LF (data not shown). We further assessed subclasses of IgG to explore differences in small subpopulations. In comparison with HC, a decreased level of IgG2 (*P* = 0.02, n = 20) was found in whole patients. Both of the IgG2 and IgG4 were found significantly decreased in SD patients (*P* = 0.002, *P* = 0.04, respectively, n = 7) (**Figures 4C, D**).

### Correlation of B Cells With CD4^+^ T Cells in Sporotrichosis Patients

**Figure 5:**
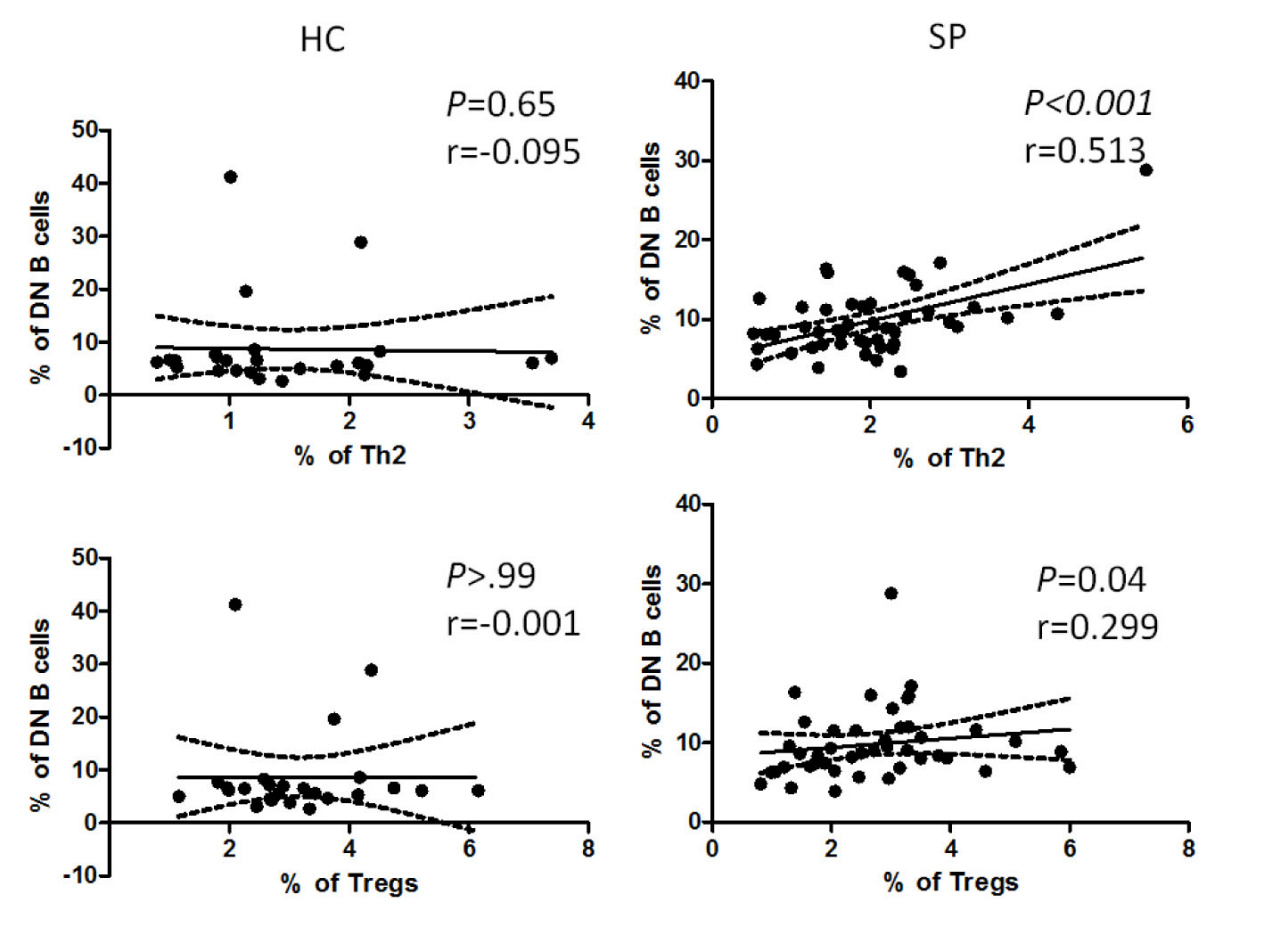
Correlation analysis between the percentage of DN B cells and that of Th2 or Tregs in patients and HC. The graphs showed linear fit in patients. The data were analyzed with Pearsoncorrelation analysis. The dotted line demonstrated a 95% confidence interval. The upper panel, the graphs for DN B cells and Th2; the lower panel, the graphs for DN B cells and Tregs. Pearson’s correlation coefficient (r) and associated *P* values are shown on each graph.

Researchers discovered that in addition to dendritic cells, B cells also take part in the differentiation of CD4^+^ T cells (30). To find out if there is any association between altered B cell subsets and CD4^+^ T cells, we further performed correlation analysis of all the altered B cell subsets (i.e. SM B cells, SM B cells and DN B cells) with Th1, Th2, Th17, Tfh, and Tregs, respectively. Interestingly, we found that the frequency of DN B cells was positively correlated with that of Th2 and Tregs (*P* = 0.01, *P* = 0.04, respectively) in patients, whereas there was no such correlation in HC (**Figure 5**). No correlation was found between the rest of altered B cell subsets and T cells in patients (data not shown).

### Altered Serum Levels of IFN-γ, IL-4, IL-17A, and TGF-β1 in Sporotrichosis Patients

To evaluate the impact of the infection in the pattern of cytokines in sera, we next performed ELISA tests of IL-4, IL-17A, IFN-γ, and TGF-β1 in patients and HC (**Figure S2A**). Compared with HC, the levels of IFN-γ (*P* = 0.04, n = 21) and IL-4 (*P* = 0.008, n = 43) were significantly increased in patients, while that of IL-17A (*P* = 0.001, n = 34) and TGF-β1 (*P* = 0.001, n = 16) were greatly reduced. However, the concentrations of IFN-γ, IL-4, and IL-17A in patients with different duration had no statistical variation from those of HC (**Figure S2B**). With regard to TGF-β1, significant lower level was found both in patients with SD (*P* = 0.04, n = 5) and those with LD (*P* = 0.005, n = 11) (**Figure S2C**).

### In Vitro Expansion of Specific CD4^+^ Teff Cells by *S. globosa*

To further understand T cell immunity against *S. globosa* infection, we established an in vitro co-culture assay to mimic in vivo interaction between PBMCs and this pathogen. PBMCs from healthy donors were cultured with/without live S. *globosa* yeast cells at different ratios (Yeasts: PBMCs, i.e. Y:P 1:1/2.5:1/5:1/10:1) for 3 days. The percentages of IFN-γ, IL-4, and IL-17 positive CD4^+^ T cells (i.e. Th1, Th2, and Th17 cells, respectively) were determined by flow cytometry (**Figure S3A**). Except the coculture at the highest Y/P ratio (10:1), all of these Teff cells’ frequencies in PBMCs were significantly elevated under the stimulation of yeast cells in a dose-dependent manner. When the load of pathogen to PBMCs reached 10:1, the proportions of these Teff cells were all comparable to that of control, however, Th1(*P* = 0.001, n = 3) and Th17 cells (*P* = 0.0005, n = 3) both notably dropped down nearly by half (**Figure S3B**).

## Discussion

We herein described a previously unrecognized Th2 inclined response in circulation of patients with sporotrichosis caused by *S. globosa*: great increases in the percentage of Th2, the ratio of Th2/Tregs as well as IL-4 level in serum in whole patients. Except for the classical role in allergic diseases, Th2 cells are also found involved in the immunity to fungal infection. Th2 response was reported to begin in later phase in systemic *S. schenckii* infected mice (27), whereas it was significantly enhanced throughout the duration in our patients. We speculated that it might be due to differences in species and strains. Till now, only one published study mentioned that Th2 response was unchanged in *S. globosa* infection in human (18). Different number of subjects and the additional investigation of Tregs in our study may contribute to the discrepancy.

So far, most studies have shown that Th2-type immunity to fungi may increase susceptibility and prolong the survival of fungal pathogens partially by inhibiting Th1 and Th17 responses in the host (31). In our study, significantly increased IL-17A^+^ Th17 in circulation was accompanied with greatly reduced serum IL-17A, indicating a defect in Th17 function. In addition, it was reported that shift of the Th1-Th2 balance toward the Th2-dominant condition induces alternatively activated macrophages and results in worsened fungal infection (32). Similarly, we also found that the ratio of IFN-γ^+^ Th1/IL-4^+^ Th2 greatly decreased in patients suggesting the shift of Th1 to Th2 pattern in *S. globosa* infection. Taken together, the enhanced Th2 response may promote pathogenesis of sporotrichosis caused by *S. globosa* partly by suppressing defensive responses of Th1 and Th17. The underlying mechanism still needs further investigations.

Tregs, the potent immunosuppressive cells, play a pivotal role in maintenance of the homeostasis of immunity. Experimental evidence reveals a complex interaction between Tregs and Teff cells in fungal infections. In vitro expanded Tregs inhibited Th1 and Th2 responses but promoted Th17 cell response to *C. albicans* (33, 34). In *S. schenckii*-infected mice, an increased Tregs response was present at early period, but declined after 21 days post infection, while *S. brasiiensis*-infected mice showed a long lasting elevated Tregs response (35). Tregs depletion in mice was accompanied with enhanced Th1/Th17 response in *schenckii*-infection (36). However, Tregs profile in human sporotrichosis is still unclear. Our data demonstrated that Tregs were significantly reduced at early stage (in 6 months) and returned to normal level in later course (more than 6 months). Whereas, TGF-β1, the most important cytokine produced by Tregs, was remarkably decreased in serum regardless of duration suggesting a dysfunction of Tregs in LD patients infected with *S. globosa*. Taken together, in early stage of *S. globosa* infection, the immune status of the host skewed to inflammatory immunity, which was further supported by enhanced Th2/Tregs and Th17/Tregs in whole patients and increases of Teff cells.

Besides Tregs, different Teff cell profiles also seem to correspond to the inoculated strain with different virulence. Among the *Sporothrix* complex, *S. brasiliensis* is the most virulent one, followed by *S. schenckii* and then *S. globosa* (7–9). In vitro, the peptides of *S. brasiliensis* could elicit a mixed Th1/Th17 cell response and the cutaneous origin of *S. schenckii* induced potent Th1 reaction (15, 37, 38). In contrast, we found unchanged Th1 and increased dysfunctional IL-17A^+^Th17 cells in addition to prominent Th2 reaction while Chen reported great decrease in both Th1 and Th17 reactions in *S. globosa* infection (18). We supposed that *S. globosa* as a low virulent fungus may favor a Th2 environment instead of intense Th1 and Th17 response in the host.

For the first time, we found greatly altered distribution of B cell subpopulations in patients infected with *S. globosa*. Our data showed substantial increased frequencies of SM B cells and DN B cells and declined USM B cells along with unchanged naïve B cells in whole patients. Similar B cell profile was found in SD compared with HC, while only DN B subset’s percentage sustained high level at later time point. Previous studies revealed that USM B cells are responsible for IgM production and SM B cells are the main subtype producing IgG (30, 39, 40). However, only levels of IgG2 and IgG4 were reduced in patients with SD, with comparable levels of total IgG and IgM in patients to those of HC. Similar to our results, Esteban found that *T. cruzi*-infected individuals had significantly lower frequencies of SM B cells with no change in the level of total IgG (41). We speculated that decreased USM B cells may be responsible for decreased IgG2 and/or IgG4 production which need further verification. Nevertheless, since we did not measure the *S. globosa* specific antibodies, we could not rule out that these altered B cell subsets may affect the production of the pathogen specific antibodies. Meanwhile, considering the unchanged Tfh cells, we presumed that there may be other factors that interact with B cell subsets in this disease.

It has been shown that antigen activated B cells can influence CD4^+^ T cell differentiation (30). In vivo and in vitro, B cells promoted Th1 and Th17 cell responses against *C. albicans* infection in IL-6 dependent manner (42, 43). In addition, Th1 differentiation was suppressed by human B cells partially via IL-10 in systemic lupus erythematosus (SLE) patients (44). Therefore, we speculated that the remodeling of B cell subsets may be associated with the disturbed T cell reaction in *S. globosa* infection. Interestingly, for the first time, we found that the greatly increased DN B cells was positively correlated with Th2 and Tregs in all patients, while no such correlation was found in HC. DN B cells were also found expanded in SLE, HIV and malaria patients, respectively (45–47), but there is no literature mentioned the relation between DN B cells and T cells till now. Although the origin and function of DN B cells remain unclear, it was suggested that DN B cells may be exhausted memory B cells (48), or precursor memory B cells that have not yet to upregulate CD27 (49), or a product which CD27 fails to upregulate appropriately (50) and the increased DN B cells in SLE patients were considered as precursors of autoantibody producing plasma cells (51). The correlation found in our study implied that DN B cells collaborated with Th2 and Tregs in the immunity against this mycotic infection in human. However, besides their function, whether DN B cells regulated Th2 and Tregs differentiation or they were expanded by Th2 and Tregs in the immunity against *S. globosa* infection still needs further investigation.

According to clinical presentations, sporotrichosis are classified into fixed cutaneous, lymphocutaneous, disseminated cutaneous, and extracutaneous sporotrichosis, among which the first two types are the most common in clinic (52). However, little is known about the underlying immunological mechanism that may contribute to various clinical manifestations. In our study, in both FF and LF patients, we found similar changes in B cell compartment and elevated ratio of Th2/Tregs; however, additionally enhanced Th2 and Th17/Tregs were found in FF patients. Involvement of Th2 immune response to fungal infection usually results in susceptibility to infection partially owing to the inhibition of protective Th1 response by IL-4 and IL-10 (53), while immunity mediated by Th17 cells is protective as evidenced by its anti-fungal role in the defense of several fungi (54–56). Although the role of Th17 function seemed to be discounted in *S. globosa* infection, the different Th17/Tregs profile in the two clinical types indicated that lack of the help of Th17 response may contribute to the fungi spreading along lymphatic vessels in lymphocutaneous form rather than besieged in a single erythematous nodule as fixed type presented.

In summary, we first demonstrated the symphony of Th2 and remarkably altered B cell subsets in patients with sporotrichosis caused by *S. globosa* and these data will help us to further understand the complex immunity against this pathogen and may provide us new strategy for clinical diagnosis and therapy of this disease and similar fungal infection.

## Data Availability Statement

The datasets presented in this study can be found in online repositories. The names of the repository/repositories and accession number(s) can be found in the article/**Supplementary Material**.

## Ethics Statement

The studies involving human participants were reviewed and approved by the First Hospital of Jilin University Research Ethics Committee. The patients/participants provided their written informed consent to participate in this study.

## Author Contributions

JZ performed the FACS analysis and analyzed the data. LY, YS, and YC performed the collection of samples. MG and RC collected the patient information. YZ and SL supervised research and reviewed data and revised the paper. JZ and YZ wrote the paper with contributions from all authors. All authors contributed to the article and approved the submitted version.

## Funding

This work was supported by the grants from the National Natural Science Foundation of China (No. 81401351 and No.81773317) and Science and Technology Development Programs of Jilin Province (20180101110JC). 

## Conflict of Interest

The authors declare that the research was conducted in the absence of any commercial or financial relationships that could be construed as a potential conflict of interest.

## Supplementary Material

Supplementary Figure 1 Immunophenotyping of circulating CD4^+^ Teff cells (Th1, Th2, Th17) in sporotrichosis patients and HC. Freshly isolated PBMCs were first stimulated with Protein Transport Inhibitor for 4 h in vitro. Cells were then subjected to CD4 staining followed by intracellular staining of IFN-γ, IL-4, IL-17A. **(A)** The subsets of CD4^+^ T cells were examined by flow cytometry. The numbers displayed are mean values of each quadrant. **(B)** Comparsion of the frequency of Teff cells in patients with HC. **(C)** Teff cell subsets are also compared between HC and patients with different duration. **(D)** The ratio of Th1/Th2 in whole patients, patients with different duration and HC. Error bars represent mean±SD. **P* < 0.05, ***P* < 0.01, ****P* < 0.001, and NS *P* ≥ 0.05.

Supplementary Figure 2 Comparison of concentration of IFN-γ, IL-4, IL-17A, and TGF-β1 in sera of sporotrichosis patients and HC. **(A)** The statistical graphs for comparison of the cytokines between patients and HC. **(B, C)** The cytokine levels are analyzed between HC and subgroups of patients with different duration. Error bars represent mean±SD. **P* < 0.05, ***P* < 0.01, and NS *P* ≥ 0.05.

Supplementary Figure 3 Coculture of PBMCs and *S. globosa* in vitro. Freshly isolated PBMCs from healthy donors were co-cultured with live *S. globosa* yeast cells at different ratios [Yeasts:PBMCs (Y:P), 1:1/2.5:1/5:1/10:1]. After 3 days co-culture, cells were restimulated with Protein Transport Inhibitor for additional 4 h and then harvested and stained as indicated. **(A)** PBMCs were fixed, permeabilized and intracellularly stained for cytokines (IFN-γ, IL-4, IL-17A) after surface staining of CD4 mAb. The flow cytometry plot presents the average proportion of Th1, Th2, and Th17 subsets in CD4^+^ T cells of infected or control. Each graph is representative of three independent experiments (n = 3). **(B)** The frequencies of CD4^+^IFN-γ^+^, CD4^+^IL-4^+^, and CD4^+^IL-17A^+^ T cells are compared between the groups with yeasts and control. Error bars represent mean±SD. **P* < 0.05, ***P* < 0.01, ****P* < 0.001, and NS *P* ≥ 0.05.
