# Supplementary figures and images for: Th2 Biased Immunity With Altered B Cell Profiles in Circulation of Patients With Sporotrichosis Caused by Sporothrix globosa
Source: Front Immunol. 2020 Nov 13;11:570888. doi: 10.3389/fimmu.2020.570888 (PMC7691245; doi:10.3389/fimmu.2020.570888)

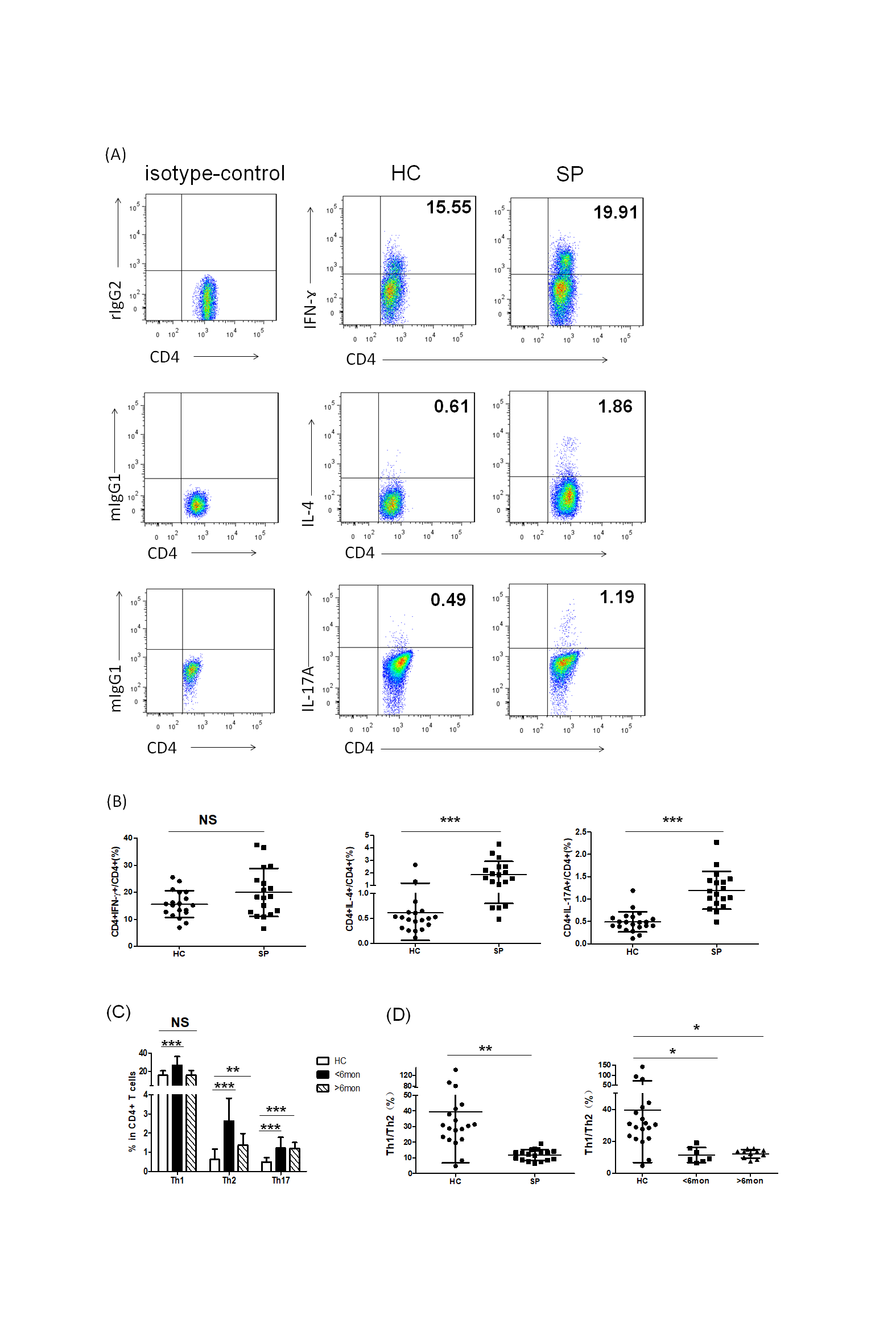

Supplement: Supplementary Figure 1 — Immunophenotyping of circulating CD4+ Teff cells (Th1, Th2, Th17) in sporotrichosis patients and HC. Freshly isolated PBMCs were first stimulated with Protein Transport Inhibitor for 4 h in vitro. Cells were then subjected to CD4 staining followed by intracellular staining of IFN-γ, IL-4, IL-17A. (A) The subsets of CD4+ T cells were examined by flow cytometry. The numbers displayed are mean values of each quadrant. (B) Comparsion of the frequency of Teff cells in patients with HC. (C) Teff cell subsets are also compared between HC and patients with different duration. (D) The ratio of Th1/Th2 in whole patients, patients with different duration and HC. Error bars represent mean±SD. *P < 0.05, **P < 0.01, ***P < 0.001, and NS P ≥ 0.05. [file Image_1.tif]

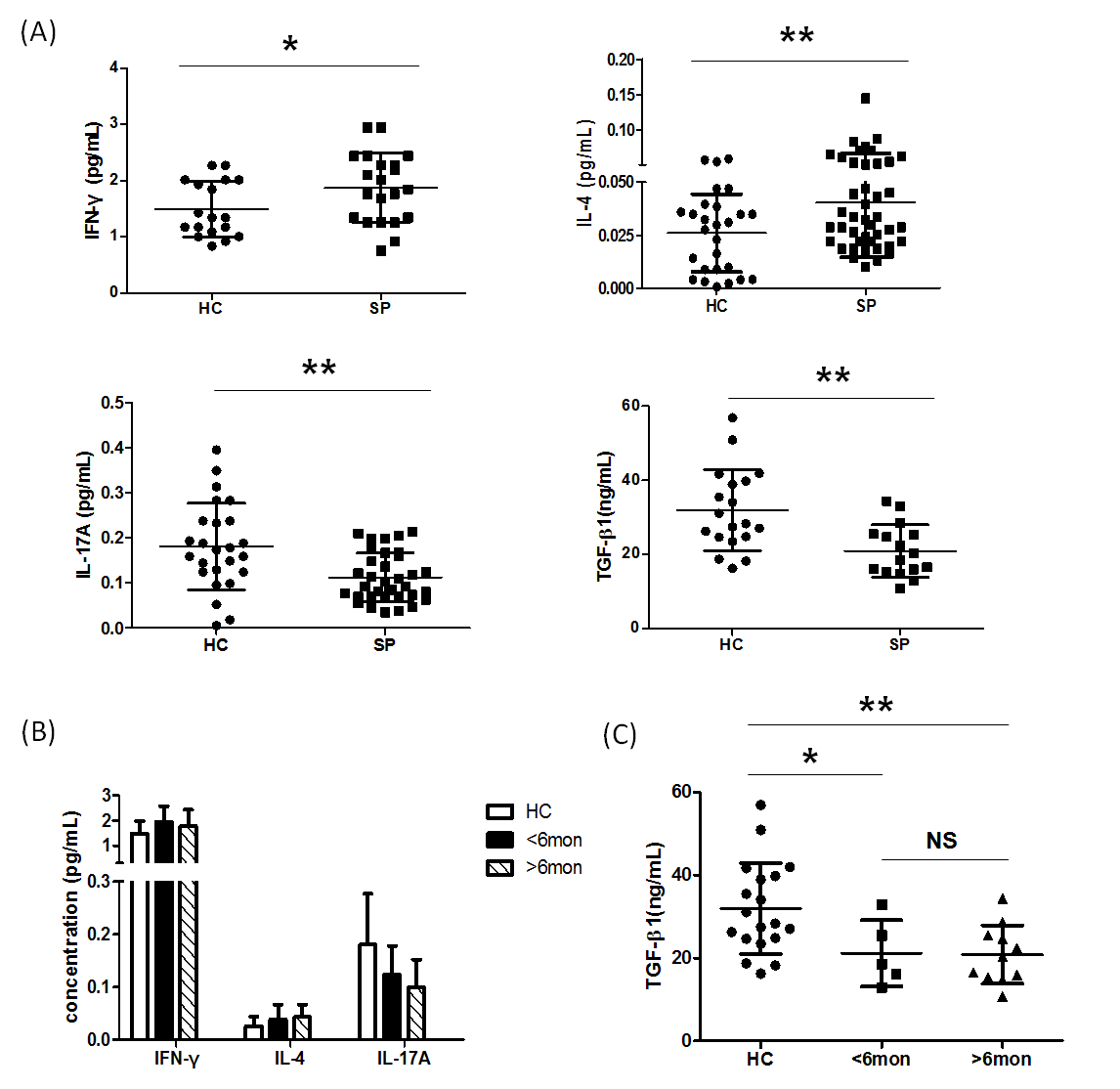

Supplement: Supplementary Figure 2 — Comparison of concentration of IFN-γ, IL-4, IL-17A, and TGF-β1 in sera of sporotrichosis patients and HC. (A) The statistical graphs for comparison of the cytokines between patients and HC. (B, C) The cytokine levels are analyzed between HC and subgroups of patients with different duration. Error bars represent mean±SD. *P < 0.05, **P < 0.01, and NS P ≥ 0.05. [file Image_2.tif]

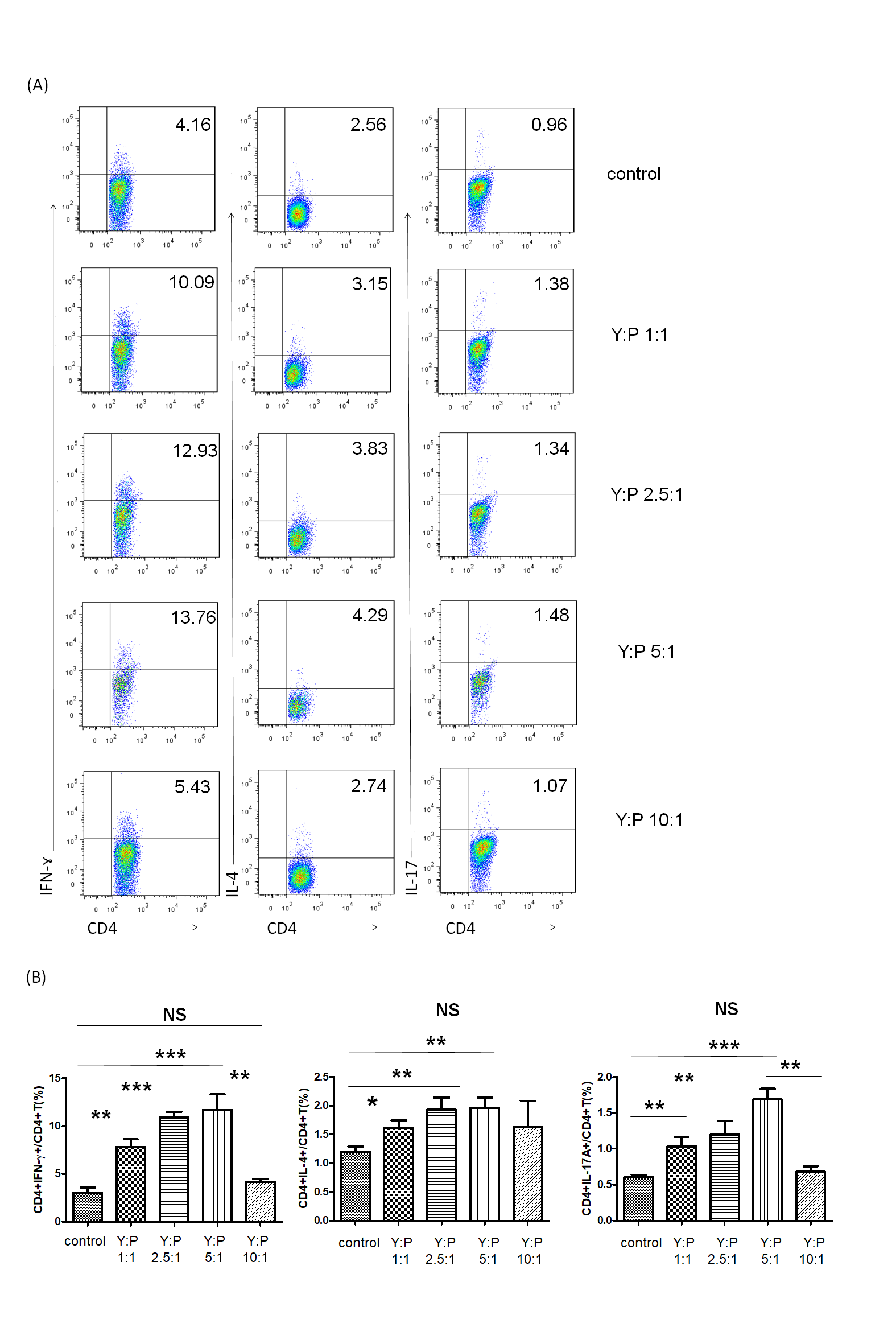

Supplement: Supplementary Figure 3 — Coculture of PBMCs and S. globosa in vitro. Freshly isolated PBMCs from healthy donors were co-cultured with live S. globosa yeast cells at different ratios [Yeasts:PBMCs (Y:P), 1:1/2.5:1/5:1/10:1]. After 3 days co-culture, cells were restimulated with Protein Transport Inhibitor for additional 4 h and then harvested and stained as indicated. (A) PBMCs were fixed, permeabilized and intracellularly stained for cytokines (IFN-γ, IL-4, IL-17A) after surface staining of CD4 mAb. The flow cytometry plot presents the average proportion of Th1, Th2, and Th17 subsets in CD4+ T cells of infected or control. Each graph is representative of three independent experiments (n = 3). (B) The frequencies of CD4+IFN-γ+, CD4+IL-4+, and CD4+IL-17A+ T cells are compared between the groups with yeasts and control. Error bars represent mean±SD. *P < 0.05, **P < 0.01, ***P < 0.001, and NS P ≥ 0.05. [file Image_3.tif]
