# Supplementary material for: Th2 Biased Immunity With Altered B Cell Profiles in Circulation of Patients With Sporotrichosis Caused by Sporothrix globosa
Source: Front Immunol. 2020 Nov 13;11:570888. doi: 10.3389/fimmu.2020.570888 (PMC7691245; doi:10.3389/fimmu.2020.570888)
Supplement: Supplementary file 4 [file Table_1.docx]

|  | **total patients (n=18)** | **Disease duration** | |  | **Presentation** | | **HC (n=20)** |
| --- | --- | --- | --- | --- | --- | --- | --- |
|  |  | duration<6 mon  (n=7; n_FF_=6, n_LF_=1) | Duration>6 mon  (n=11; n_FF_=10, n_LF_=1) |  | Fixed form (n=16; n_SD_=6, n_LD_=10) | lymphocutaneous  form (n=2; n_SD_=1, n_LD_=1) |  |
| **Age**  **(years, mean±SD)** | 55±14  10/8  7.9±7.3 | 62±10  4/3  3.5±1.5 | 51±16  6/5  10.7±8.8 |  | 55±15  8/8  8.2±8.1 | -  -  - | 47±7  10/10  ̶ |
| **Female/Male** |  |  |  |  |  |  |  |
| **Average duration (months, mean±SD)** |  |  |  |  |  |  |  |

**Table S1. Demographic characteristics of sporotrichosis patients and healthy control**

**Note: these patients were enrolled from 2020.07.10 for supplementary experiments.**
